# Adaptive Beam Divergence Control to Mitigate Scintillation Effect Caused by Pointing Error in Vertical FSO Transmissions

**DOI:** 10.3390/s23115045

**Published:** 2023-05-24

**Authors:** Hye-Min Park, Young-Jin Hyun, Sang-Kook Han

**Affiliations:** Department of Electrical and Electronic Engineering, Yonsei University, Seoul 03722, Republic of Korea

**Keywords:** free-space optical communications, adaptive beamwidth, atmospheric turbulence, free-space optical vertical link

## Abstract

Free-space optical (FSO) communication has been studied for next-generation network systems. Because an FSO system establishes point-to-point communication links, maintaining alignment among the transceivers is a critical challenge. In addition, atmospheric turbulence causes significant signal loss in FSO vertical links. Even in clear weather conditions, transmitted optical signals suffer significant scintillation losses due to random variations. Thus, the effect of atmospheric turbulence should be considered in vertical links. In this paper, we analyze the relationship between pointing error and scintillation from the aspect of beam divergence angle. Furthermore, we propose an adaptive beam that optimizes its divergence angle according to the pointing error between the communicating optical transceivers to mitigate the effect of scintillation due to pointing error. We performed a beam divergence angle optimization and compared it with adaptive beamwidth. The proposed technique was demonstrated using simulations, which revealed an enhanced signal-to-noise ratio and the mitigation of the scintillation effect. The proposed technique would be useful in minimizing the scintillation effect in vertical FSO links.

## 1. Introduction

Non-terrestrial networks (NTNs) were developed as next-generation wireless backhaul systems to provide network services everywhere. Because of several advantages, such as higher bandwidth, greater security, and immunity to electromagnetic interference, and network flexibility, free-space optical (FSO) communication has been thoroughly studied for implementation in NTNs [1]. Recently, unmanned aerial vehicle (UAV)-assisted FSO communication has been actively studied to connect regions hundreds of kilometers away. High-altitude platform (HAP) UAVs establish FSO-based backhaul networks to resolve atmospheric fading and connect regions hundreds of kilometers away.

Because an FSO system establishes point-to-point communication links, maintaining alignment among the transceivers is a critical challenge [2]. The mechanical vibrations in UAVs and building sway cause pointing errors. Therefore, appropriate pointing, acquisition, and tracking (PAT) procedures require directing the laser beam from the transmitter to the receiver and maintaining the alignment between them during communication. There are two ways to maintain line of sight in FSO communication: transmitting a broad beam for pointing error and increasing the transmitter power using a narrow beam. However, transmitting a broad beam results in geometric loss, and using a narrow beam causes pointing loss [3]. Hence, considering the abovementioned trade-off and deciding on a proper beam divergence angle on the basis of the signal-to-noise ratio (SNR) or capacity of the FSO link is a well-known technique [4,5,6,7].

**Figure 1:**
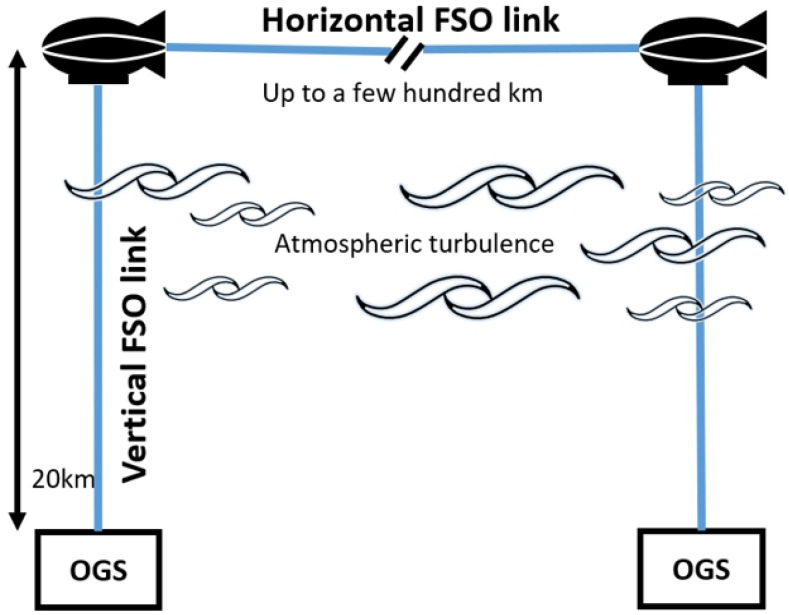
UAV-based vertical and horizontal FSO links.

In the case of a horizontal link, a UAV altitude of 20 km is considered to mitigate the turbulence effect, as shown in Figure 1. However, atmospheric turbulence causes significant signal loss in an FSO vertical link for the optical ground station (OGS) [8]. Even in clear weather conditions, transmitted optical signals suffer significant scintillation losses due to random variations in the atmospheric refractive index [9,10]. This effect is induced by the variation in the refractive index of the atmosphere, which can cause the light to bend and scatter as it travels through the air. Consequently, it causes random fluctuations in received signal intensity. Thus, the effect of atmospheric turbulence should be considered, and scintillation mitigation methods have been studied accordingly [11,12,13].

The present investigation is based on two aspects: first, a rapid increase in scintillation index with a slight increment in pointing error [14]; second, less affection by scintillation in a larger beam width due to the broad beam propagation from spherical wave to plane wave [15]. The narrow beam propagates as a Gaussian beam from the aspect of beam divergence angle. By contrast, the broad beam propagation is from the Gaussian beam to a spherical wave. Accordingly, the wider the divergence angle, the less is the affection caused by scintillation. The scintillation effect increases exponentially as the number of pointing errors increases. Therefore, the use of wider beams is proposed to reduce the scintillation effect when pointing errors constantly occur.

In this paper, we propose an adaptive beam that adjusts its divergence angle according to the pointing error between the communicating optical transceivers to mitigate the effect of scintillation due to pointing error. Adaptive beam divergence control has been investigated for several purposes [16,17,18,19,20]. In the work of Harada et al. [16], the beam was controlled for a variable distance, and Mai and Kim attempted to improve link availability or save transmitter power while preserving target link availability [17]. Mai and Kim also reported a beaconless PAT system [18] and adaptive beam control to mitigate the effects of angle-of-arrival fluctuation and pointing error [19,20]. However, most of the research focuses on the horizontal link without considering scintillation. Scintillation causes errors in the received signal, leading to degradation of communication performance, making it insufficient to establish seamless communication systems. Therefore, we analyzed the relationship between pointing error and scintillation from the aspect of beam divergence angle. Furthermore, we verified that a slight increment in pointing error causes a rapid increase in the scintillation index, and a large beam divergence angle can reduce the effect of scintillation. Hence, the proposed adaptive beam adapts its divergence angle according to the pointing error between the communicating optical transceivers to reduce the effect of scintillation due to pointing error.

## 2. FSO Vertical Channel Model

In this study, the FSO channel was based on the Hufnagel–Valley (H–V) atmospheric model, and far-field diffraction uplink propagation with a Gaussian beam was assumed [21]. Because downlink is less affected by scintillation, uplink was modeled in this study, which considered the effect of scintillation. In addition, background noise, amplified spontaneous emission (ASE) beating noise, and dark current noise, which significantly affect the FSO channel, were considered [22].

### 2.1. Atmospheric Turbulence Channel

The H–V model is most widely used to represent the vertical atmospheric turbulence. The $C_{n}^{2}\left(h\right)$ model to describe the varying strength of optical turbulence as a function of altitude *h* is

(1) $$C_{n}^{2}\left(h\right)=0.00594{(\nu /27)\;}^{2}{{(\;10}^{-5}\;h)}^{2}\exp\;(-h/1000)+2.7\times 10^{-16}\;\exp\;(-h/1500)+C_{n}^{2}\left(0\right)\;\exp\;(-h/100),$$

where ν is the root-mean-square (rms) wind speed, and $C_{n}^{2}\left(0\right)$ is the refractive index structure at the ground in $m^{-\frac{2}{3}}$. In this study, we assumed ν = 21 m/s and $C_{n}^{2}\left(0\right)$ as

(2) $$C_{n}^{2}\left(0\right)=1.7\times 10^{-13}m^{-\frac{2}{3}}.$$

These values correspond to the H–V 5/7 model and weak turbulence assumption.

The normalized variance of the scintillation index is defined as

(3) $$\sigma_{I}^{2}=\frac{<{Ι}^{2}>-<{Ι>}^{2}}{<{Ι>}^{2}}=\frac{<{Ι}^{2}>}{<{Ι>}^{2}}-1,$$

where *I* is the irradiance, and < > denotes an ensemble average. Following the approach of Miller et al. [23], the irradiance variance can be expressed as follows:

(4) $$\sigma_{I}^{2}\left(r,L\right)=\;\sigma_{I}^{2}\left(0,L\right)+\;\sigma_{I,r}^{2}\left(r,L\right),$$

where r denotes the positions at the transverse planes of the configuration and the spatial frequency spaces, and *L* is the link distance. The first term in Equation (4) is the on-axis normalized variance, and the second term is the fundamental of the finite beam scintillation index, commonly known as the radial component. The total uplink scintillation index is defined as

(5) $$\sigma_{I}^{2}\left(r,L\right)=\;8.702k^{\frac{7}{6}}{\left(H-h_{0}\right)}^{\frac{5}{6}}{sec}^{\frac{11}{6}}\left(\xi \right)\times \left[\mu_{1}+1.667\frac{\mu_{1}\Lambda^{\frac{5}{6}}\alpha^{2}{\left(H-h_{0}\right)}^{2}{sec}^{2}\left(\xi \right)}{w_{0}^{2}\left(\Omega_{0}^{2}+\Omega^{2}\right)}\right],$$

where k=2π/λ, and λ,ξ, *H*, $h_{0},$ and $w_{0}$ are the wavelength, zenith angle, UAV altitude, height of the OGS, and collimated beam width, respectively. We assumed the wavelength to be 1550 nm, the zenith angle and height of the OGS as 0, and the collimated beam width as 10 cm. A collimated beam is characterized by $\Omega_{0}=1$, $\Omega =2L/kw_{0}^{2}$, with receiver beam parameters θ= $\Omega_{0}/(\Omega_{0}^{2}+\Omega^{2})$ and Λ = $2L/kw^{2}$, where $w=w_{0}{\left(\Omega_{0}^{2}+\Omega^{2}\right)}^{\frac{1}{2}}$ is the diffractive beam radius at the receiver. The angles in radians that determines the radial distance of the beam from the optical axis is given by α=r/L, where r is defined as the radial distance from the beam center line.

The quantity $\mu_{1}$ is the integral defined as [24]

(6) $$\mu_{1}=\;Re\int_{h_{0}}^{H}C_{n}^{2}\left(h\right)\left\{{\left(1-\frac{h-h_{0}}{H-h_{0}}\right)}^{\frac{5}{6}}\left[\Lambda \left(1-\frac{h-h_{0}}{H-h_{0}}\right)+i{\left(1+\theta -\frac{\theta h-h_{0}}{H-h_{0}}\right)}^{\frac{5}{6}}\right]-\Lambda^{\frac{5}{6}}{\left(1-\frac{h-h_{0}}{H-h_{0}}\right)}^{\frac{5}{6}}\right\}dh.$$

### 2.2. Beam Divergence Angle Optimization

As we assume far-field diffraction propagation with a Gaussian beam, the radial term of the electric field of the Gaussian beam is defined as [25,26,27,28]

(7) $$E\left(r,z\right)=\exp\left(\frac{{-r}^{2}}{{w\left(z\right)}^{2}}\right),$$

where ***r*** is the pointing error, and $w\left(z\right)$ is the radius at which the field amplitudes fall to 1/e of their axial values at the plane *z* along the beam, defined by

(8) $$w(z)=w_{0}\sqrt{1+{\left(\frac{z}{z_{R}}\right)}^{2}},$$

where $z_{R}$ is the Rayleigh range given by

(9) $$z_{R}=\frac{\pi w_{0}^{2}}{\lambda }.$$

The received optical power can be calculated as

(10) $$P_{Rx}=\;P_{Tx}G_{Tx}G_{Rx}E^{2}L_{s},$$

where $P_{Tx}$ is the transmitter power assumed to be 1 W, $G_{Tx}={8/(w\left(z\right)/L)}^{2}$ is the transmitter gain, $G_{Rx}=\pi^{2}D^{2}/\lambda^{2}$ is the receiver gain, where D = 10 cm is the aperture diameter, and $L_{s}={\left(\lambda /4\pi L\right)}^{2}$ is the space loss.

**Figure 2:**
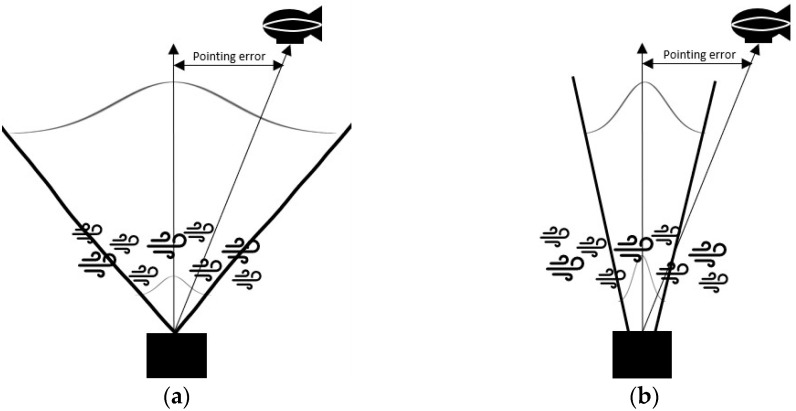
Interrelationship among scintillation, pointing error, and beam divergence angle: (**a**) A broad beam to compensate for pointing error; (**b**) A narrow beam to increase the optical power.

Because the beam divergence angle is related to transmitter gain, the optimal beam size should be decided. The transmitter gain is considered to satisfy the required SNR for the high-data-rate transmission. The wider the beam divergence angle, the lower is the transmitter gain. In addition, the beam divergence angle is determined from the interrelationship between the beam divergence angle and pointing error. As shown in Figure 2a, when the OGS transmits a broad beam to compensate for pointing error, the broad beam results in a geometric loss. Conversely, transmitting a narrow beam to increase the transmitter power results in pointing loss, as shown in Figure 2b. Furthermore, the scintillation is related to the beam divergence angle.

From the aspect of beam divergence angle, a narrow beam propagates as a Gaussian beam. However, a broad beam propagates from a Gaussian beam to a spherical wave. Moreover, the Gaussian beam is known to be most affected by scintillation, followed by spherical waves and plane waves [29]. Thus, the greater the divergence angle, the less is the affection caused by scintillation.

**Figure 3:**
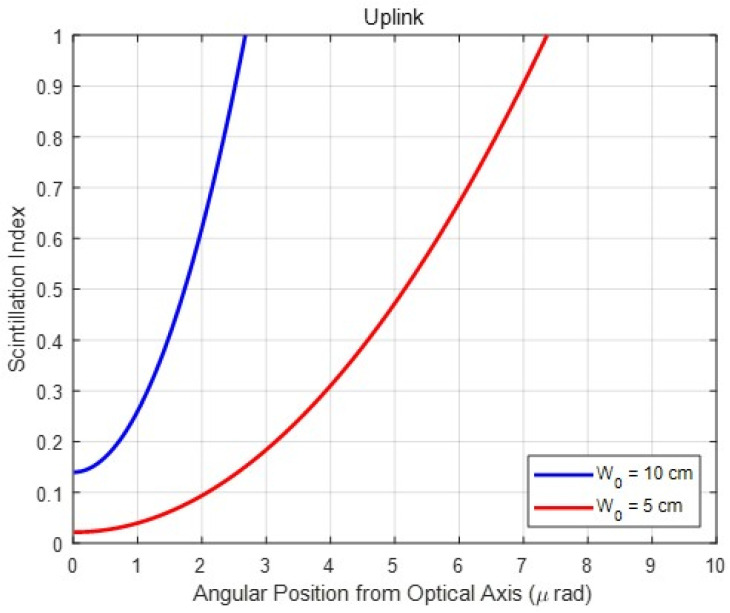
Scintillation index for pointing error when two collimated beam sizes, 10 and 5 cm, are set for a link distance of 20 km.

Furthermore, the pointing errors are also related to the scintillation index. As shown in Figure 3, two beam widths (10 and 5 cm) are collimated at the transmitter. The *X*-axis represents a function of an angular receiver position from the optical axis representing the pointing error, and the *Y*-axis denotes the scintillation index. Thus, Figure 3 presents the scintillation index according to pointing errors. According to Andrews et al. [14], a slight increment in pointing error induces a rapid increase in the scintillation index. The link distance was at a satellite in geostationary orbit at 38,500 km. A similar result is obtained when the distance is assumed to be 20 km as shown in Figure 3.

The *X*-axis represents the pointing error, and the scintillation index increases rapidly with a slight increase in pointing error. In other words, as more pointing errors occur, the scintillation effects increase exponentially. However, the scintillation effects are minor without pointing error because the Gaussian beam is propagated to spherical waves at the on-axis. Furthermore, regardless of the beam divergence angle, the scintillation effect is negligible in the case of downlinks. This is because the atmospheric turbulence affects the beam after being sufficiently approximated as a spherical wave.

In addition, as shown in Figure 3, the larger collimated beam size is more affected by the off-axis scintillations compared to the smaller beam. This is due to a narrow divergence angle for propagating the giant collimated beam and a broad divergence angle for propagating the smaller beam. The scintillation affects the narrow beam more owing to the pointing error. Therefore, when pointing errors occur frequently, wider beams are proposed to mitigate the scintillation effect in an FSO vertical uplink channel. However, the transmitter gain is insufficient when a wide beam is constantly utilized to reduce the effect of scintillation. Therefore, the beam must be adjusted adaptively according to the pointing error.

## 3. Adaptive Beamwidth Control

Several studies have been conducted on adapting to control the beam divergence angle, for example, using a component of an FSO terminal with a basic design that uses a common optical path and an established adaptive beam control [30]. Furthermore, a method of adaptively adjusting the beam divergence angle using a variable focal lens has been studied [18,19,20]. In this study, we added a beam divergence controller to the existing method to control the beam divergence angle adaptively.

**Figure 4:**
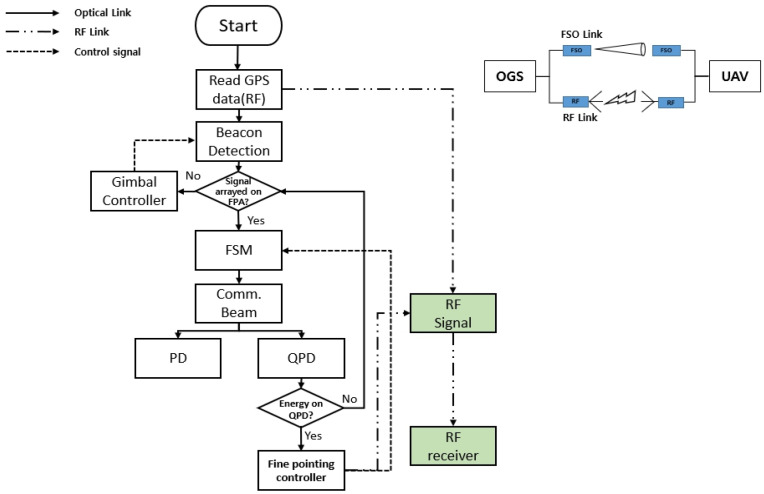
Adaptive beam control flowchart.

Figure 4 shows the proposed adaptive beam control flow. FSO and radio frequency (RF) links are assumed to be utilized concurrently in the OGS and UAV. First, the receiver starts coarse pointing based on RF Global Positioning System (GPS) information. Then, the beacon controls the gimbal according to the information generated in the focal plane array (FPA). When the beacon is aligned with the FPA, a narrow communication beam is sent to start fine-pointing. When the communication beam gets aligned, a fast-steering mirror (FSM) starts fine-tuning using the information in the quadrant photodiode (QPD). When data transmission begins, the communication beam is divided between the photodiode (PD) and QPD, which receive and track the data, respectively. The QPD continuously reads the position of the received beam, calculates the pointing error, and sends it to the transmitter using the RF feedback link. If the QPD does not receive the communication beam, the FPA will check whether the beacon signal is detected, and the gimbal will be controlled for alignment. Once the beacon is aligned, the communication beam is aligned again.

**Figure 5:**
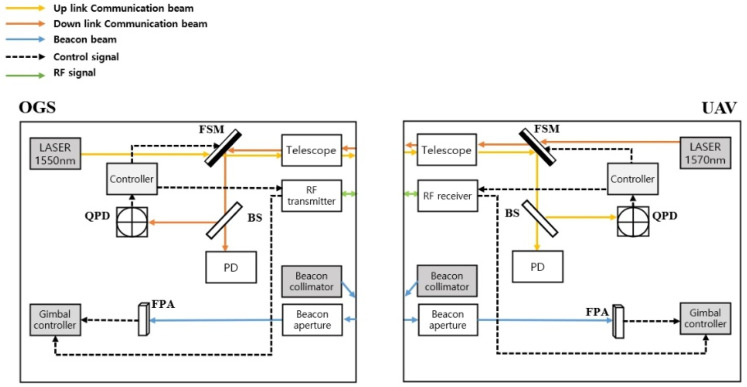
PAT schematic for OGS-to-UAV communication.

Figure 5 shows the schematics of the PAT procedures with adaptive beam control for OGS-to-UAV communication. Both the OGS and UAV use a beacon beam, a separate telescope that exchanges a communication beam, and an aperture that exchanges a beacon beam. To prevent interference between uplink and downlink, wavelengths of the communication beam were taken as 1550 and 1570 nm, respectively. On the basis of the GPS information received from RF signals, the gimbal controls the body, which can be oriented in a wide range. Then, the beacon is sent in that direction, and the beacon adjusts the gimbal more finely according to the information arrayed to the FPA. Once the arraying procedure is complete, a communication beam is sent after passing the FSM. It will then be divided into a PD and a QPD by beam splitter (BS). The PD receives data and controls the FSM using the information in the QPD so that the beam can be accurately oriented. The QPD sensor data are used as a beam spot monitor, and the pointing error can be used to calculate the position of the beam on the QPD. The estimated pointing error is exchanged using the RF feedback link. Finally, the beam divergence controller controls the transmit beam angle according to the estimated pointing error.

## 4. Simulations and Results

To examine transmission performance using simulation results, the vertical FSO system presented in Table 1 was considered. We set the vertical link distance to 20 km from an OGS to a UAV operating under minimal wind speed using the Bufton wind model [31]. The refractive structure at the ground was estimated to be 1.7 $\times 10^{-13}$ $m^{-\frac{2}{3}}$ to examine the effect of increased strong turbulence on the vertical FSO link. The transmitter power was assumed as 30 dBm (1 W), and the modulation format of the FSO system was set to PAM4 to support high-data-rate transmission. The standard deviation of the pointing error was assumed to be 5 μrad for fine-pointing [32], and the noise power was a value in which background noise, ASE beating noise, and dark current noise were all considered.

We considered the relationship between scintillation, pointing error, and beam width to obtain the optimal beam width. The pointing errors were produced following the Rayleigh distribution beam widths. From the pointing errors, scintillation indexes were generated according to log-normal random distributions. The received optical power $P_{Rxopt}$ considered for these relationships is

(11) $$P_{Rxopt}=\;P_{Rx}L_{ptg}L_{SI},$$

where $L_{ptg}$ is the pointing error loss given by

(12) $$L_{ptg}=\exp\left(-8{\left(\frac{\theta_{ptg}}{\theta^{\frac{1}{e^{2}}}}\right)}^{2}\right),$$

where $\theta_{ptg}$ is angle of pointing error. The $L_{SI}$ is scintillation loss according to log-normal random distributions about $\theta^{\frac{1}{e^{2}}}$ and $\theta_{ptg}$.

By assuming the noise relative to the received power to be the same, the bit error rate (BER) was calculated using the PAM 4 BER formular, and the beamwidth was selected with a minimum BER average. In other words, the optimal beam size was determined as a beam width with a minimum BER value calculated as

(13) $$\underset{\theta^{1/e^{2}}}{\min}\left(\frac{1}{T}\int_{0}^{T}{BER}_{PAm4}(t)dt\right)=\underset{\theta^{1/e^{2}}}{\min}\left(\frac{1}{T}\int_{0}^{T}\frac{1}{2}erfc\left(\frac{P_{Rxopt}(\theta^{\frac{1}{e^{2}}},\theta_{ptg}(t))}{4P_{N}}\right)dt\right),$$

where *T* is a long enough period of time. 

For the adaptive beam, a pointing error is calculated by QPD at a specific time. The beam divergence controller controls the transmit beam angle according to the estimated pointing error $\theta_{ptg}$.

(14) $$\underset{\theta^{1/e^{2}}}{\min}\left({BER}_{PAm4}\right)=\underset{\theta^{1/e^{2}}}{\min}\left(\frac{1}{2}erfc\left(\frac{P_{Rxopt}(\theta^{\frac{1}{e^{2}}},\theta_{ptg})}{4P_{N}}\right)\right).$$

**Figure 6:**
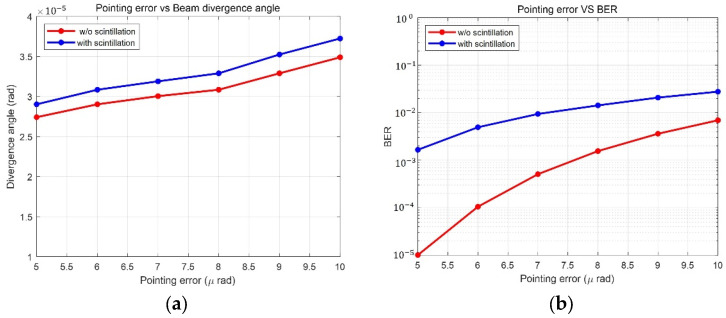
Difference between cases with and without scintillation in terms of (**a**) divergence angle according to the pointing error; (**b**) BER according to the pointing error of each divergence angle.

Figure 6 shows the difference between the cases with and without scintillation. Figure 6a shows the relationship between beam divergence angle and pointing error. The markers on the graph represent the optimal beam width at each pointing error. A wider beam divergence angle is required when the pointing error increases from 5 to 10 μrad. The optimal beam divergence angle ranges from 27 to 35 μrad without scintillation and 29 to 37 μrad with scintillation. In this simulation, the fixed beam divergence angle was determined to be 29 μrad considering 5 μrad pointing errors. Because this beam width is represented when the BER is optimized, Figure 6a does not clearly indicate the scintillation effect. In Figure 6b, the BER according to the pointing errors shows the scintillation effect more clearly. In Figure 6b, the BER plot shows a significant difference under the same assumption. The BER that satisfies $10^{-5}$ becomes less than $10^{-3}$ with the consideration of scintillation. Thus, the scintillation causes a considerable reduction in BER.

**Figure 7:**
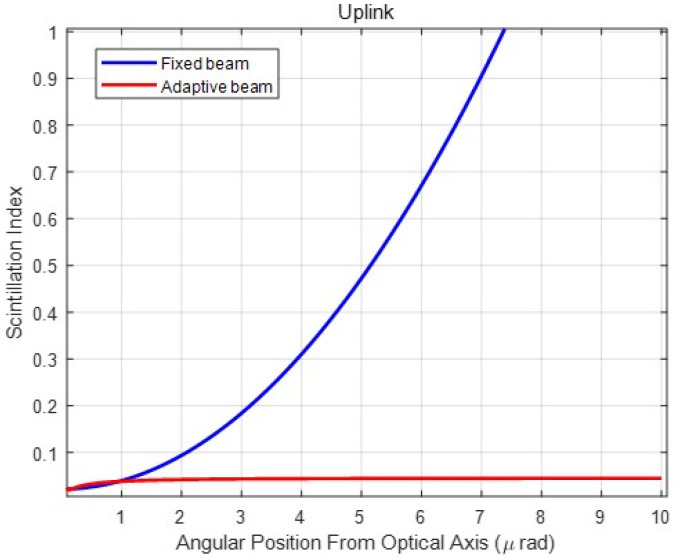
Scintillation index according to the pointing error: comparison of fixed and adaptive beams.

Figure 7 shows the scintillation index according to the pointing error in both fixed and adaptive beams. As previously mentioned, the fixed beam was optimized with a beam width with a minimum BER, and the adaptive beam was widened according to the pointing error. In the case of the optimal fixed beam, the scintillation index increased rapidly with a slight increase in pointing error. However, the scintillation effect was constant for the pointing error when the beam was controlled adaptively. The adaptive beam control is based on calculated pointing errors. A larger number of pointing errors cause a greater scintillation effect, which can be reduced by expanding the beam divergence angle. Compared to Figure 3, we proved that the proposed adaptive beam reduced the scintillation effect caused by pointing error.

**Figure 8:**
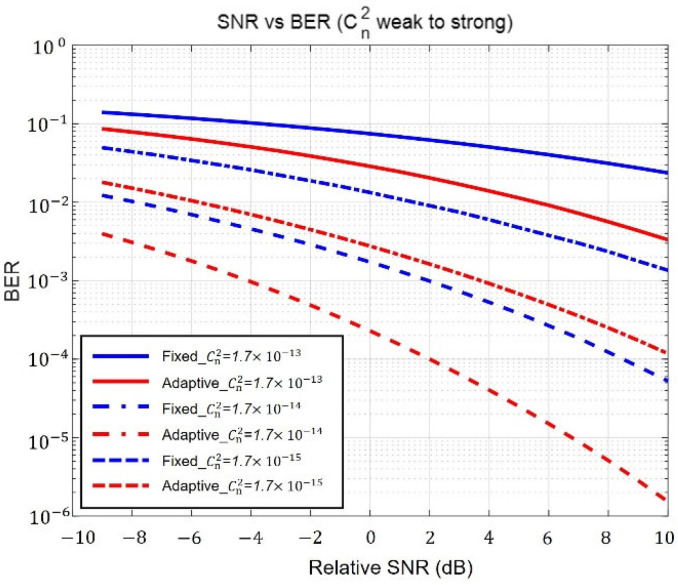
BER and SNR comparison of fixed and adaptive beams according to the strength of ground turbulence.

Figure 8 presents the BER relative to SNR in both fixed and adaptive beams based on the strength of ground turbulence from top to bottom in order. The relative SNR is as a function of the variation in noise power. Although Figure 8 is not a graph that directly reflects the pointing error result, the improvement in adaptive performance is still apparent. In weak turbulence ($1.7\times 10^{-15}$), the relative SNR performance of the adaptive beam improved by 6 dB compared to the fixed beam. In moderate turbulence ($1.7\times 10^{-14}$), it improved by 5 dB, and in strong turbulence ($1.7\times 10^{-13}$), it improved by 4 dB. Thus, as ground turbulence strengthens, adaptive beam performance is enhanced compared to a fixed beam. This phenomenon is due to two main reasons. First, the fixed beam was optimized to a broad beam in strong turbulence and a narrow beam in weak turbulence. Because the narrow fixed beam could not sufficiently compensate for pointing errors in weak turbulence, the BER was degraded. In addition, the BER performance of a narrow beam for weak turbulence was further impaired owing to the increase in scintillation caused by the pointing error. The second reason is the flexibility of an adaptive beam. The fixed beam is a narrow beam, whereas the adaptive beam is broadened according to the pointing error. Consequently, the adaptive wide beam in weak turbulence is less affected by scintillation. As mentioned above, the scintillation affects the wide beam more than the narrow beam.

**Figure 9:**
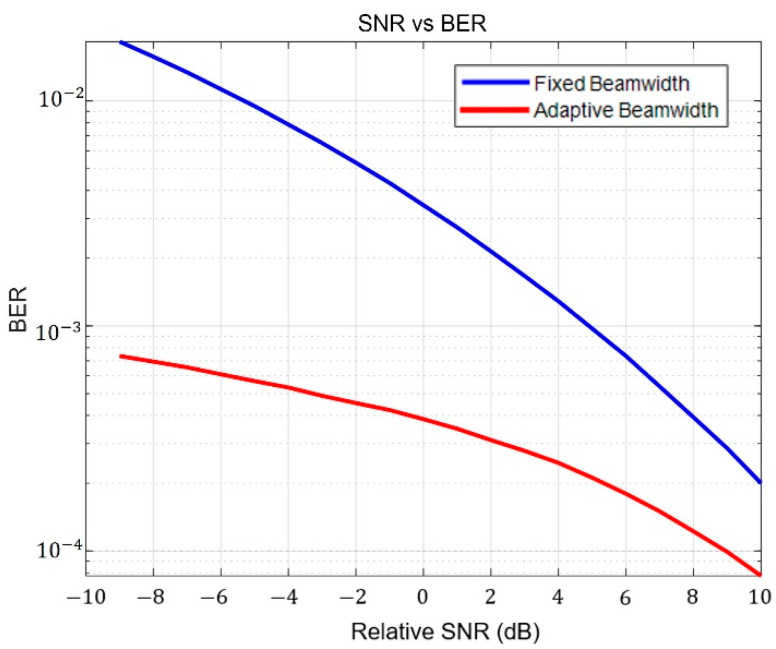
BER and SNR comparison of fixed and adaptive beams using power allocation to enhance the SNR.

Because the fixed beam is optimized to a minimum BER, the beam is fixed to the broad beamwidth to compensate for the pointing error. Therefore, in a comparison based on SNR, the difference is less significant. However, the use of additional power allocation schemes can increase SNR performance in adaptive beam control systems. Figure 9 shows the SNR–BER relationship in fixed and adaptive beamwidth using power allocation. The received power decreases because of broadening to compensate for the pointing error. To enhance the SNR, the power secured in the narrow beam was allocated to the broad beam. Thus, the result showed significant differences in SNR performance in fixed and adaptive beams. In addition, the data rate can be enhanced in an adaptive modulation method by adjusting the beam adaptively. The use of an ultimate beamwidth according to the pointing error will save power, and the reserved power enables transmitting signals using a high-order modulation technique. The adaptive modulation according to received power enhances the data rate, with the power secured using the narrow beam for a high modulation order.

## 5. Conclusions

In summary, we propose an adaptive beam divergence angle control method to mitigate the effect of scintillation caused by pointing errors. We verified the performance of the proposed technique using FSO channel modeling. In addition, we analyzed the relationship between pointing error and scintillation, which is necessary for determining the optical beam divergence angle from the aspect of beam divergence angle. In addition, theoretical evaluations proved that the suggested method mitigates scintillation when a pointing error exists. Adaptive beams caused an improvement in SNR compared to fixed beams, and it was shown that applying the secured power could lead to more progress. Furthermore, the data rate could be enhanced by concurrently using adaptive beam divergence control and modulation. The proposed technique would help minimize the scintillation effect in vertical FSO links, which incur considerable losses owing to random fluctuations in the refractive index of the atmosphere. Furthermore, the proposed technique can be used to configure a system in an environment with frequent occurrence of pointing errors by moving along a specific trajectory because the UAV cannot hover. We believe the proposed technique would help next-generation industry backhaul networks considering a combination of pointing error and scintillation.

## Figures and Tables

**Table 1:** Vertical FSO link parameters.

Parameter (Symbol)	Value
Wavelength (λ)	1550 nm
Transmitter power ($P_{Tx}$)	30 dBm
Modulation format	PAM 4
Aperture diameter (D)	10 cm
Link distance (L)	20 km
RMS wind speed (v)	21 m/s
Refractive index structure on the ground ($C_{n}^{2}$)	${1.7\times 10}^{-13}m^{-\frac{2}{3}}$
Standard deviation of pointing jitter (***r***)	5 μrad
Target bit error rate (uncoded)	${1\times 10}^{-9}\;({1\times 10}^{-3}$)
Noise power ($P_{N}$)	${2\times 10}^{-5}$ W

## Data Availability

Not applicable.
